# Task Dependent Effects of Head Orientation on Perceived Gaze Direction

**DOI:** 10.3389/fpsyg.2018.02491

**Published:** 2018-12-06

**Authors:** Tarryn Balsdon, Colin W. G. Clifford

**Affiliations:** ^1^School of Psychology, University of New South Wales, Sydney, NSW, Australia; ^2^Laboratory of Perceptual Systems and Laboratory of Cognitive Neuroscience, Department of Cognitive Studies, École Normale Supérieure, PSL University, CNRS, Paris, France

**Keywords:** gaze perception, head orientation, dual-route model, social vision, response bias

## Abstract

The perception of gaze direction involves the integration of a number of sensory cues exterior to the eye-region. The orientation of the head is one such cue, which has an overall repulsive effect on the perceived direction of gaze. However, in a recent experiment, we found the measured effect of head orientation on perceived gaze direction differed within subjects, depending on whether a single- or two-interval task design was employed. This suggests a potential difference in the way the orientation of the head is integrated into the perception of gaze direction across tasks. Four experiments were conducted to investigate this difference. The first two experiments showed that the difference was not the result of some interaction between stimuli in the two-interval task, but rather, a difference between the types of judgment being made across tasks, where observers were making a directional (left/right) judgment in the single-interval task, and a non-directional (direct/indirect gaze) judgment in the two-interval task. A third experiment showed that this difference does not arise from observers utilizing a non-directional cue to direct gaze (the circularity of the pupil/iris) in making their non-directional judgments. The fourth experiment showed no substantial differences in the duration of evidence accumulation and processing between judgments, suggesting that observers are not integrating different sensory information across tasks. Together these experiments show that the sensory information from head orientation is flexibly weighted in the perception of gaze direction, and that the purpose of the observer, in sampling gaze information, can influence the consequent perception of gaze direction.

## Introduction

Human observers are especially good at judging the direction of another’s gaze, with empirical measures suggesting judgments of gaze direction can be about as accurate as human visual acuity would permit (Cline, 1967; Jenkins and Langdon, 2003). This ability is important for social interactions, where information about where someone else is looking gives observers insight into the contents of other’s thoughts. It has been suggested that the human eye evolved to facilitate accurate gaze perception, with increased contrast between the white sclera and the dark pupil, in comparison to other primates (Kobayashi and Kohshima, 1997, 2001). This allows for increased accuracy in judging gaze direction based on the iris eccentricity (Anstis et al., 1969; Emery, 2000) and the contrast polarity of the dark pupil against the white sclera (Ricciardelli et al., 2000; Sinha, 2000). In addition to cues from the eye region, observers integrate a number of other cues exterior to the eye region, such as the orientation of the head, and the emotional expression of the looker. Some authors have therefore suggested that perceived gaze direction may be computed in a more holistic manner (Tanaka and Farah, 1993; Tanaka and Simonyi, 2016) in order to estimate the overall attentional direction of the looker, or by integrating information in a hierarchical manner, whereby more global information (such as head orientation) is utilized when local information (such as the relative position of the pupil) is unavailable (Perrett et al., 1985, 1992).

When the head is oriented directly toward the observer, the observer can judge the direction of gaze based on the relative position of the pupil within the eye opening, for example, a leftward pupil indicates leftward gaze. However, the task of judging gaze direction becomes more complicated if the head is not oriented directly toward the observer. When viewing real human faces, Gibson and Pick (1963) found observers’ perception of gaze was biased in the opposite direction to head orientation, and similar results have been found with tightly controlled realistic face stimuli (Anstis et al., 1969; Otsuka et al., 2014, 2015). In contrast, the Wollaston effect (Wollaston, 1824) shows gaze direction tends to be perceived in the same direction as head orientation in artificial stimuli where the same eye-region is placed in the context of differently oriented heads. Similar results have been found when cartoon faces are moved laterally within a cartoon head, simulating the face eccentricity changes that coincide with head turn (Todorovic, 2009). Thus, the orientation of the head can have both a repulsive and an attractive effect on perceived gaze direction.

**FIGURE 1:**
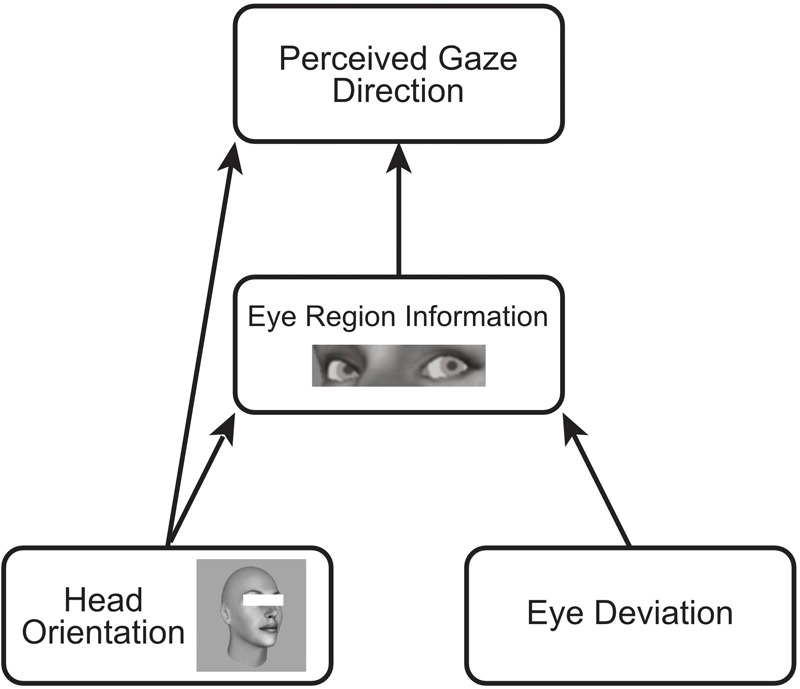
Schematic of the dual-route model of the influence of head orientation on perceived gaze direction. The orientation of the head has an indirect effect on perceived gaze direction via changes in the eye-region. This indirect effect repulses the perceived gaze direction away from the orientation of the head as the relative amount of visible sclera on either side of the pupil changes in a similar manner as when gaze is averted in the opposite direction. Head orientation also has a direct effect on perceived gaze direction, acting as a coarse scale spatial cue to gaze direction, attracting perceived gaze in the same direction as head orientation.

The dual route model, illustrated in Figure 1, offers a functional account of these two seemingly opposite effects of head orientation on perceived gaze direction (Otsuka et al., 2014, 2015, 2016). The indirect repulsive effect results from the effect of head turn on the eye region information: As the head rotates, the information from the eye region projected to the observer changes in a number of ways, for example, the projected shape of the eyes change, some of the eye may be obscured by the bridge of the nose, and importantly, the amount of visible sclera changes such that, as the head rotates to the right there is increased sclera visible to the right of the pupil, in the same manner as if gaze had shifted to the left in a direct facing head. In this way, the information within the eye region changes with head rotation in a similar manner as if gaze had shifted in the opposite direction to the head, such that the observer may perceive gaze to be directed more in the opposite direction of head rotation. This indirect repulsive effect is mitigated somewhat by a direct attractive effect of head orientation on perceived gaze direction. The orientation of the head acts as a coarse scale spatial cue to gaze direction, causing observers to perceive gaze more in the same direction as head orientation. These cues to gaze direction are weighted differently, resulting overall in a stronger repulsive effect of head orientation on perceived gaze direction in naturalistic faces, which becomes stronger if the observer is shown only the eye region (thereby weakening the information contributing to the direct attractive effect; Otsuka et al., 2014, 2015).

**FIGURE 2:**
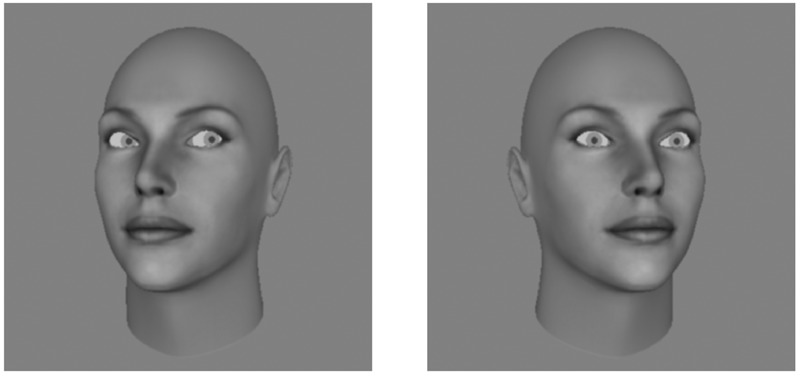
Example stimuli. The left image shows a head rotated to the left by 15 degrees, whilst the right image shows a head rotated to the right by 15 degrees. In both stimuli gaze is oriented 10 degrees to the right, however, the typical viewer will not perceive the gaze directions as exactly the same. Rather, gaze in the leftward oriented head will be perceived as more rightward than gaze in the rightward oriented head.

The overall repulsive effect of head orientation on perceived gaze direction (in naturalistic faces) has been measured in a number of ways. Most previous experiments have used a single-interval design, where the observer is shown a single stimulus and asked to make some judgment about the direction of gaze. Gibson and Pick (1963) asked observers to decide if gaze was directed at them or not, Otsuka et al. (2015) asked observers to judge whether gaze was directed left, right, or direct, with respect to themselves, and Anstis et al. (1969) and Otsuka et al. (2016) asked observers to report the exact direction of gaze, for example, by orienting a pointer in the same direction as the gaze of the stimulus. These experiments produce similar results, using different stimuli and different response types, all measuring an overall repulsive effect of head orientation on perceived gaze direction. This repulsive effect can also be demonstrated simply by examining the example stimuli in Figure 2, where the same gaze deviations in differently oriented heads are not perceived as gazing in the same direction. Rather, the typical viewer will perceive gaze in the leftward oriented head as more rightward than the same degree of gaze offset in the rightward oriented head.

In a recent experiment, Balsdon and Clifford (2017) found a far weaker repulsive effect of head orientation on perceived gaze direction than has previously been measured. The experiment compared a commonly used single-interval design with a two-interval design, to investigate the use of the two-interval design as a method of eliminating the possibility of response bias from measures of perceived gaze direction. In the single-interval design, a single stimulus is shown to the observer and they are asked to make a judgment about the direction of gaze. In the two-interval design the observer is shown two stimuli (one after the other) and asked which of the two has more direct gaze. Because the order of the stimuli in the two-interval task is counterbalanced, a tendency to make a certain response does not correspond to reporting a particular stimulus, whereas, in the single-interval design observers may easily develop a tendency to report, for example, leftward gaze, when presented with a rightward oriented head. The two-interval design thereby minimizes response bias, and thus would be more appropriate for comparing the effect of head orientation across populations that may differ systematically in their response biases, such as in patients with schizophrenia (Bentall and Slade, 1985; Brébion et al., 1998). The measured effect in the single-interval task was similar to previous measures, with an overall ‘weighting’ of head orientation of -0.25, corresponding to a repulsive effect. In the two-interval task the measured weighting of head orientation was 0.07 on average, corresponding to a slight overall attractive effect.

The single- and two-interval tasks are proposed to measure the same perceptual effect, and thus any difference between the measurements made across the two tasks would normally be attributed to response bias in the single-interval task. The results of the two-interval task would therefore suggest that the measured repulsive effect in the single-interval task is merely the result of observers adopting a tendency to respond that gaze is oriented in the opposite direction to the head. This suggestion is untenable for two reasons. First, the repulsive effect can be directly observed by examining example stimuli as in Figure 2. If the true perceptual effect were actually attractive, then gaze would be perceived more in the same direction as head orientation, yet the reader should find that gaze direction in the leftward oriented head appears more rightward than in the rightward oriented head. Second, previous experiments have used different response types, so if the repulsive effect were entirely due to the tendency to make a particular response, this should differ with the required response. Furthermore, there is no theoretical basis (nor, to our knowledge, empirical evidence) for a systematic response bias to be implemented in tasks that require observers to report the perceived gaze direction on a scale, such as with the pointer judgment used by Otsuka et al. (2016), and the protractor adjustment used by Anstis et al. (1969). The difference between the single- and two-interval tasks must therefore be explained by something other than response bias. The four experiments presented in this manuscript extend and replicate this work by systematically examining the differences between these two tasks and what causes the differences in the behavioral responses.

The first experiment sought to examine whether any difference in the stimulus presentation across the two tasks could account for the differences in the measured effects of head orientation on perceived gaze direction. Of particular concern was the presentation of two oppositely oriented heads in quick succession in the two-interval task. In the single-interval task, the order of stimuli was randomized, and there was a slightly longer duration between stimulus presentations, as observers were responding after each stimulus. In the two-interval task, the presentation of the first head could alter the perception of the second head by means of fast adaptation, such that the second head appears more repulsed from the first: if the perceived orientation of the head were exaggerated in this way, without altering the information within the eye region, then the measured effect on perceived gaze direction would be more attractive, as was observed in Balsdon and Clifford (2017). If this were the case, then presenting two heads of the same orientation would result in the opposite bias as is measured when oppositely oriented heads are presented. Another possible confound is that, when the head is oriented, one eye is closer to the observer than the other, depending on head orientation. Evidence suggests that observers rely more on the information from the closer eye (Noll, 1976), so the presentation of opposing heads may present observers with the problem of having to switch their attention between eyes, or rely on weaker information from the further eye in one of the intervals. These possibilities would also be mitigated in a two-interval design where the same head orientation is presented across both intervals. To further eliminate any possibility of interactions between stimuli, the following experiment also modified the stimulus presentation procedure of Balsdon and Clifford (2017) to include 500 ms of spatially filtered noise after each stimulus presentation, and add jitter to the stimulus presentation location from stimulus to stimulus.

An additional between subjects condition was added to the experimental procedure, in which participants were presented with the same stimuli as in Balsdon and Clifford (2017), but were only shown the eye region of the stimuli. In these stimuli the direct attractive effect of the head is reduced, whilst the indirect repulsive effect is maintained, resulting in an overall greater repulsive effect of head orientation on perceived gaze direction (Otsuka et al., 2014, 2015). Another possible difference between tasks is that, in the single-interval task, observers may be able to mostly ignore the orientation of the head and focus on the relative iris-eccentricity, which would drive a stronger repulsive effect, whereas, in the two-interval task, observers’ attention is drawn to the head because two oppositely oriented heads are presented in quick succession. If the difference between tasks resulted from some difference in the way observers were attending to the surrounding head, then there should be no difference between tasks when observers are presented with only the eye-region of the stimuli.

## Experiment 1

The methods are fundamentally the same as those presented in Balsdon and Clifford (2017). Changes to these methods are explicitly stated in the full description below.

### Methods

#### Participants

Participants were recruited from the UNSW first year psychology participation scheme after the study was given ethical approval by the UNSW human research ethics committee, which adheres to the declaration of Helsinki. A total of 49 participants were recruited and randomly allocated to one of two conditions (whole-head and eye-region) such that 24 participants completed the whole-head condition, and 25 completed the eye-region. All participants gave written informed consent to participating. After applying the exclusion criteria as detailed in the analysis section, 20 participants in each condition remained in the analysis.

#### Apparatus

Stimuli were presented using MATLAB (Mathworks) and the Psychtoolbox extensions (Brainard, 1997; Pelli, 1997; Kleiner et al., 2007) on a 32″ Display++ LCD monitor (Cambridge Research Systems, Rochester, United Kingdom) with a refresh rate of 120 Hz and resolution 1920×1080, with a gray background, mean luminance 60 cd/m^2^. Participants sat 57 cm from the screen with their chin on a chin rest.

#### Stimuli

Four gray-scale faces (two male and two female), with cropped hair and neutral expressions, were created with Daz software^1^. Observers were presented with two versions of each face, one rotated 15° to the right and the other 15° to the left, an example is shown in Figure 2. To control for any effect of stimulus asymmetry, on half the trials, a rightward oriented face was presented by vertically flipping the leftward oriented face and similarly, a leftward oriented face was presented by vertically flipping a rightward oriented face. Eye deviation was manipulated by replacing the original eyes with realistic counterparts that could be moved according to precise angular coordinates. The whole-head stimuli were presented to fit in approximately 400×400 pixels, subtending approximately 14×14 degrees of visual angle, and are the same as used in Balsdon and Clifford (2017). In the eye-region condition (not included in Balsdon and Clifford, 2017), the same stimuli were presented, but only the region around the eyes was drawn on the screen, a rectangle of 130×30 pixels centered on the bridge of the nose. Stimuli were presented within a 400 ms temporal window against a gray background, with a 100 ms raised cosine ramp at onset and offset temporally bordering 200 ms at full contrast. To prevent motion cues, the position of the stimuli on the screen was jittered randomly within a region up to 15×15 pixels from the screen center on each presentation. Each stimulus was followed immediately by a 500 ms presentation of spatially filtered noise (where the spatial amplitude spectrum matched that of the face stimuli). These precautions were not in place in Balsdon and Clifford (2017).

#### Procedure

Observers completed two tasks: the single-interval task, and the two-interval task. The single-interval task was conducted exactly as in Balsdon and Clifford (2017), with the exception of the modifications to stimulus presentation outlined above (jitter in the stimulus position, and the additional noise mask following stimuli). On each trial observers were shown a single stimulus and were asked to respond as to whether the gaze of the stimulus was directed to the right or to the left of them. Responses were entered by pressing ‘1’ for left, and ‘2’ for right, on a standard QWERTY keyboard. Observers were presented with 11 eye deviations ranging from -10° to +10° in steps of 2° for each head orientation, with 14 presentations of each eye-deviation/head orientation, making a total of 308 trials.

The two-interval task was modified to contain two conditions: opposite-head and same-head conditions, which were pseudo-randomly intermixed within each block. The opposite-head condition was the same as presented in Balsdon and Clifford (2017) (with the exception of the changes to stimulus presentation outlined above). On each trial, observers were presented with two stimuli (of the same face identity) in succession (with each stimulus followed by 500 ms of noise). Observers were asked to respond as to which interval contained gaze that was more directed at them. Responses were entered by pressing ‘1’ for first interval and ‘2’ for second interval. In the opposite-head condition one interval contained a rightward oriented head and the other contained a leftward oriented head (the order of which was chosen at random). Observers were presented with 11 pairs of eye-deviations, which differed by -10° to +10°, in steps of 2°, relative to base deviations of ±5° (separate trials). With 14 repetitions of each of these trials, there were 308 trials in the opposite head condition. In the same-head condition the head orientations of stimuli in both intervals were either leftward oriented or rightward oriented. The eye-deviations of these stimuli were the same as in the opposite-head condition, except that they were presented relative to a base deviation of -5° in one stimulus and +5° in the other of the pair (the order of which was counterbalanced across trials). Again, 14 repetitions of these trials were used, making 308 trials in the same-head condition, totaling 616 intermixed trials in the two-interval task. Both the single- and the two-interval tasks were completed in a single session of less than 1 h. The order of the tasks was randomized across participants.

#### Analysis

The analysis was conducted in the same manner as Balsdon and Clifford (2017).

In the single interval task, logistic functions were fit to each participant’s proportion of ‘rightward’ responses, and the point of subjective equality (PSE, the gaze deviation at which half the fitted responses were rightward) was taken as the gaze deviation corresponding to subjectively direct gaze. The influence of head orientation was then taken as half the difference between the PSEs for the leftward and rightward oriented heads.

In the two-interval task, opposite-head condition, logistic functions were fit to each participant’s proportion of trials where the stimulus with a rightward oriented head was chosen. The PSE was the point at which gaze was perceived as equally direct (or equally averted) between the two heads, and the average of the PSEs for base deviations of -5° and +5° was taken as the influence of head orientation on perceived gaze direction. A similar analysis was conducted on the same-head condition, except the PSEs were now calculated for pairs of leftward and rightward heads separately, and the influence of head orientation on perceived gaze direction was taken as half the distance between the PSEs for the leftward and rightward head trials.

Participant’s data were excluded from further analysis based on two criteria. First, if the inverse slope of the logistic function exceeded the range of deviations tested, indicating that their responses did not vary systematically with the gaze deviations presented. Second, if the calculated PSE was outside the range of deviations presented, since an accurate measure of the PSE would not be possible in this case. Further inspection of the data indicated that some participants excluded for these reasons appeared to be responding to the orientation of the head rather than the direction of gaze, whilst others appeared non-compliant with experimental instructions.

All statistical analyses were carried out on the measures of the influence of head orientation on perceived gaze direction, as calculated from the PSEs. However, we also present the calculated weighting of head orientation for comparison with previous experiments (Otsuka et al., 2014). The relative weighting (*w*) of head orientation is calculated such that:

wH+(1−w)E=0

Where *H* is the orientation of the head and *E* is the gaze deviation corresponding to perceptually direct gaze. Rearranging this equation gives:

$$w=\frac{-E}{H-E}$$

The relative weighting of head orientation is independent of the degree of head rotation (assuming a linear relationship) such that measures can be compared across experiments that employ stimuli of differing head orientations.

### Results

**FIGURE 3:**
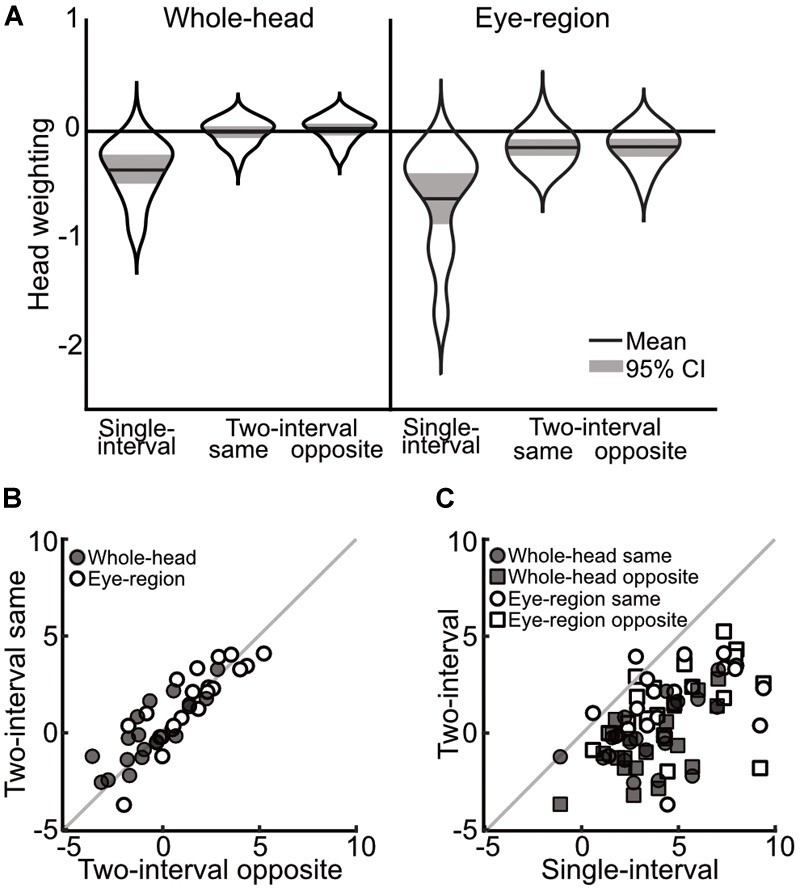
Comparison of measurements across single-interval, two-interval opposite head, and two-interval same-head tasks, from Experiment 1. **(A)** Distribution of measures of the weighting of head orientation in each task. The width of each ‘violin’ corresponds to the probability density at each head weighting. The mean is shown by the solid black line and can be compared to 0, which shows veridical perception. The shaded area is the 95% confidence interval. A negative head weighting indicates a net repulsive effect. **(B)** Individual measurements of the influence of head orientation on perceived gaze direction in the opposite heads two-interval task compared to the same heads two-interval task. The whole-head condition is shown with filled markers and the eye-region condition shown with open markers. **(C)** Individual measures in the single- and two-interval tasks. The whole-head condition is shown in filled markers and the eye-region condition shown in open markers. Square vs. Circular markers are used for the opposite- and same-head tasks, respectively. The gray lines show equality. Measurements in **(B,C)** are from the raw PSEs (in units of degrees), and thus a positive value indicates a net repulsive effect.

A 3×2 mixed ANOVA, with task (single-interval, two-interval same-head, and two-interval opposite-head) as a within subjects measure and condition (whole-head and eye-region) as a between subjects measure, showed a significant effect of task [*F*(1.35,51.31) = 96.63, *p* < 0.001, $\eta_{p}^{2}$ = 0.72] and a significant effect of condition [*F*(1,38) = 10.62, *p* = 0.002, $\eta_{p}^{2}$ = 0.22], but no significant interaction [*F*(1.35,51.31) = 0.64, *p* = 0.473, $\eta_{p}^{2}$ = 0.02]. Within subjects comparisons include a Greenhouse-Geisser correction for violating the assumption of sphericity [*χ*^2^(2) = 24.26, *p* < 0.001]. The main effect of task was clearly driven by the difference in the measures from the single-interval task compared to the two-interval opposite-head task (mean within-subject difference = 3.75° ± 0.68° 95%CI) and the two-interval same-head task (mean within-subject difference = 3.44° ± 0.71°), compared to which the difference between the same- and opposite-head two-interval tasks was miniscule (mean within-subject difference = -0.31° ± 0.34°). These measures, transformed into weightings of head orientation, are shown in Figure 3A.

In order to examine the relationship between measures across tasks, tests of the correlations between the tasks were carried out by fitting a line (*y* = *mx* + *b*) that minimizes the perpendicular distance to each point (the influence of head orientation on perceived gaze direction measured in one condition compared to another), and taking the slope of this line as the correlation. The significance of the correlations was tested using a non-parametric bootstrap analysis, where a sample of data was drawn from the full data (with replacement) to match the original experiment, and assessing the slope of the best fitting line over 1,000 iterations of this procedure. The strongest correlations were found between the two-interval tasks (opposite vs. same heads; whole-head, *m* = 0.91, *p* < 0.002; eye-region, *m* = 1.02, *p* < 0.002), as can be seen from the closeness of the data to the line of equality in Figure 3B. The single-interval and two-interval tasks also showed some correlation (Figure 3C) in the whole-head condition (opposite-head *m* = 0.75, *p* = 0.024; same-head *m* = 0.62, *p* = 0.034), and for the same-head two-interval task in the eye-region condition (*m* = 0.54, *p* = 0.022), but was not significant in the opposite-head two-interval task (*m* = 0.53, *p* = 0.108).

Measurements in the whole-head condition were very similar to those presented in Balsdon and Clifford (2017), despite the changes to stimulus presentation that eliminated motion cues and the possibility of interactions between stimuli. The measurements from the single-interval eye-region condition are also in line with previous experiments (Otsuka et al., 2014, 2015, 2016). The two-interval eye-region condition measured, on average, an overall repulsive effect of head orientation on perceived gaze direction. A stronger repulsive effect in the eye-region condition is expected under the dual-route model, as the removal of the surrounding head weakens the direct attractive effect of the head, thereby resulting in a stronger net repulsive effect. Although the two-interval task shows measurements that could be suggestive of a very minimal overall effect of head orientation on perceived gaze direction in the whole-head condition, there is a net repulsive effect measured when only the eye-region is shown to observers. The two-interval task therefore displays a similar pattern of measurements as the single-interval task (a stronger repulsive effect in the eye region condition) that is consistent with the dual-route model, the difference is rather that the overall repulsive effect is measured as weaker in the two-interval task (or that the overall attractive effect is measured as stronger) both in the whole-head and in the eye-region conditions. In both the whole-head and the eye-region condition there was little evidence for a difference between the opposite- and same-head trials in the two-interval task, and indeed the measures were tightly correlated. There was a large significant difference between the measurements from the single- and two-interval tasks in both the whole-head and eye-region condition, and although the measures were still correlated (observers who displayed a stronger repulsive effect in one task also tended to display a stronger repulsive effect in the other task), these correlations were weaker, and in one case, not significant. The results of this experiment therefore rule out the possibility that any superficial difference in the nature of stimulus presentation across tasks could account for the differences in measurements.

Another possible difference between the tasks is the specific decision observers are making. In the single-interval task, observers are asked to decide whether the eyes are looking to the left or to the right of them, whereas, in the two-interval task, observers are making a decision concerning how direct gaze is. There is some experimental evidence for a difference between reporting direct gaze compared to making judgments about the relative direction of gaze. For example, Seymour et al. (2017) found that the “direct gaze bias” measured in patients with schizophrenia, where patients report a wider range of gaze deviations as being directed at them when asked if they are “being looked at,” is not apparent when patients are asked to judge whether gaze is directed “left, right, or straight ahead.” Patients with Schizophrenia therefore had trouble adopting an egocentric perspective, making judgments about gaze direction relative to themselves, but displayed no difference from typical observers when adopting an allocentric perspective.

Experiment 2 was designed to begin addressing this question of whether observers draw on different sensory cues to make different judgments about the direction of another’s gaze. A new group of participants were recruited to complete the single-interval and two-interval tasks, in addition to a new version of the single-interval task where the judgment matched that of the two-interval task – they were asked to respond as to whether gaze was directed at them or not. If the difference between the single- and two-interval tasks is the result of the different types of judgments required, then there will be no difference between the single- and two-interval tasks when observers are asked to make the same type of judgment in each task, that is, a judgment about the directness of gaze.

## Experiment 2

### Methods

Methods were the same as in Experiment 1, with the following exceptions.

#### Participants

A new group of participants were drawn from the same pool as Experiment 1. A total of 49 participants completed the experiment, with 24 in the whole-head condition, and 25 in the eye-region condition. All participants gave written informed consent to participating. After applying the same exclusion criteria as Experiment 1, 20 participants remained in the analysis for each condition.

#### Procedure

The two-interval task was altered to contain only the opposite-head trials, as it was originally designed, and participants were asked to judge which interval contained the more direct gaze. Participants were also asked to complete two versions of the single-interval task. The left/right (l/r) version asked for the same judgment as previously, responding ‘1’ if gaze was directed to the left of them, and ‘2’ if gaze was directed to the right of them. The direct yes/no (y/n) version asked participants to judge whether gaze was directed at them or not, and participants were instructed to press ‘1’ if the eyes were looking directly at them, and ‘2’ otherwise. The stimuli in both single-interval tasks were exactly the same, and all stimuli were presented in the same manner as Experiment 1, with a 500 ms presentation of a noise patch following each stimulus, and the location of the stimulus jittered randomly on each presentation to be within 15×15 pixels of screen center. The three tasks were completed in a single experimental session of less than 1 h, and the order of the tasks was randomized across participants.

#### Analysis

**FIGURE 4:**
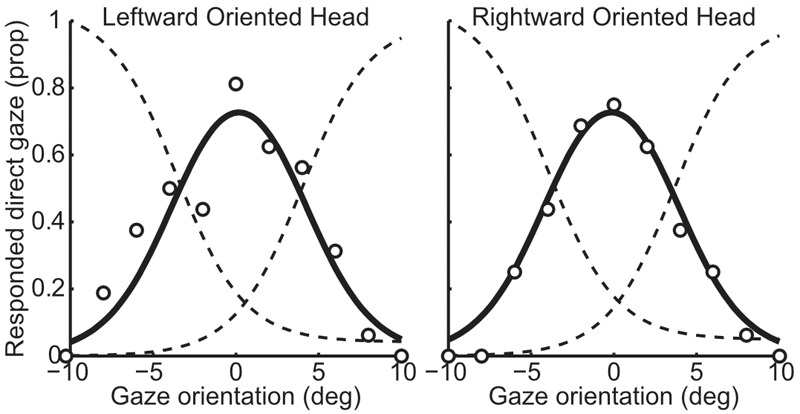
Example data from one participant in the whole-head condition from the single-interval y/n task. The left panel shows responses to the leftward oriented head and the right panel shows responses to the rightward oriented head. The circles show the actual proportion of responses whilst the solid black line shows the fitted proportion. The solid black line is calculated as the difference between the two logistic functions shown in dotted lines (the leftward curve is reversed for demonstration), which were fitted by minimizing the sum of squared error of the data points from the black line.

Data processing was conducted in the same manner as Experiment 1 for the single-interval l/r task and the two-interval task. A slightly more complex approach was required for the single-interval y/n task: The proportion of ‘direct’ responses at each gaze deviation was fit with the difference from two logistic functions (one that would correspond to increasing ‘leftward gaze’ responses with more leftward gaze, and one that would correspond to increasing ‘rightward gaze’ responses with more rightward gaze, had the participants been asked to identify the direction of indirect gaze), as shown in Figure 4. The logistic functions were constrained to have the same slope and be equidistant from the peak of the proportion of direct responses. Four parameters could therefore describe the proportion of direct responses in both head orientations: the peak of the proportion of direct responses in each head orientation (two parameters), the distance between the means of the two logistic functions, and the slope of the logistic functions. Four logistic functions were defined from these parameters, all sharing the same slope, with the means calculated from the parameters for the peak of the proportion of direct responses and the distance between the means. The difference between pairs of logistic functions was then fit to the proportion of direct responses in each head orientation. The peak of the proportion of direct responses was taken as the gaze deviation perceived to be most direct. Analogous to the single-interval l/r task, the influence of head orientation in the single-interval y/n task was calculated as half the difference between the gaze deviation perceived to be most direct in the leftward and rightward oriented heads.

### Results

**FIGURE 5:**
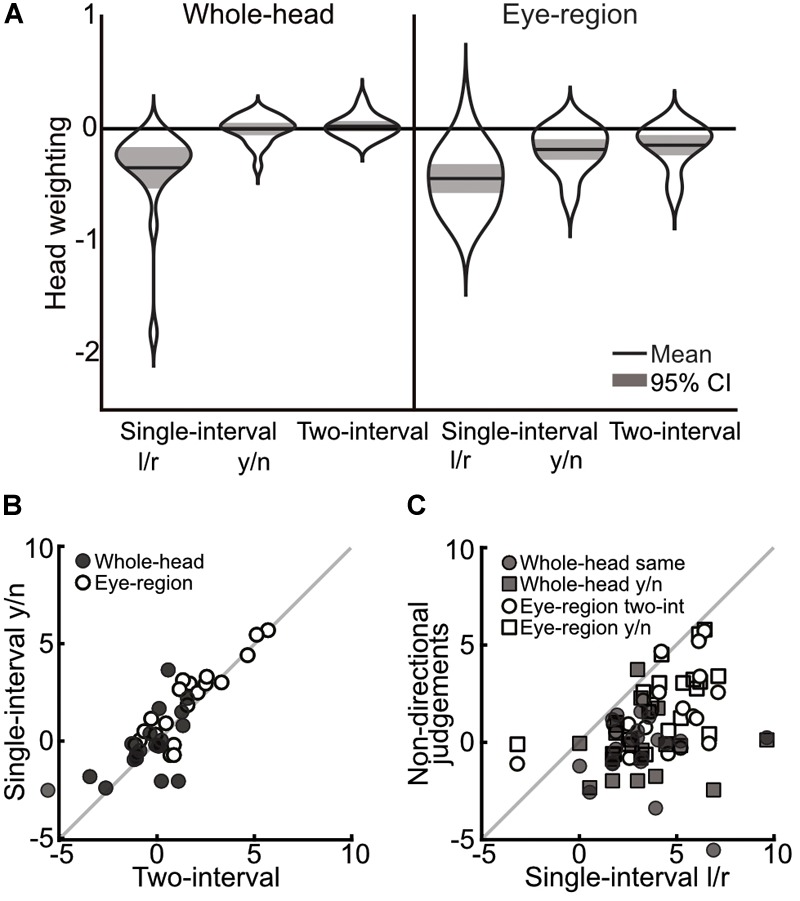
Comparison of measurements across single-interval l/r, single-interval y/n, and two-interval tasks, from Experiment 2. **(A)** Distribution of measures of the weighting of head orientation in each task. The width of each ‘violin’ corresponds to the probability density at each head weighting. The mean is shown by the solid black line and can be compared to 0, which shows veridical perception. The shaded area is the 95% confidence interval. A negative head weighting indicates a greater repulsive effect. **(B)** Comparison of individual measures of the influence of head orientation on perceived gaze direction in the single-interval y/n and the two-interval task. The whole-head condition is shown with filled markers, and the eye-region condition is shown with open markers. **(C)** Comparison of the single-interval l/r task with the tasks in which a non-directional judgment was made (the single-interval y/n and the two-interval tasks). The whole-head condition is shown in filled markers and the eye-region condition shown in open markers. Square vs. Circular markers show the single-interval y/n and two-interval tasks, respectively. The gray line shows equality. Measurements are from the raw PSEs, and thus a positive value indicates a net repulsive effect.

A 3×2 mixed ANOVA with task (single-interval l/r, single-interval y/n, and two-interval) as a within subjects factor, and condition (whole-head and eye-region) as a between subjects factor, revealed a significant main effect of task [*F*(1.35,51.4) = 52.258, *p* < 0.001, $\eta_{p}^{2}$ = 0.58], and a significant effect of head condition [*F*(1,38) = 12.377, *p* = 0.001, $\eta_{p}^{2}$ = 0.25], but no significant interaction [*F*(1.35,51.4) = 2.238, *p* = 0.133, $\eta_{p}^{2}$ = 0.06]. Within subjects comparisons include a Greenhouse-Geisser correction for violating the assumption of sphericity [*χ*^2^(2) = 24.12, *p* < 0.001]. The difference between the single-interval y/n and the two-interval tasks was negligible (mean within subjects difference = 0.39 ± 0.39 95% CI) compared to the difference between the single-interval l/r task and the two-interval task (mean within-subjects difference = 3.19 ± 0.85) and with the single-interval y/n task (mean within subjects difference = 2.80 ± 0.77). The overall means, transformed into head weightings, are presented in Figure 5A.

Tests of correlations were carried out as in Experiment 1, with the strongest correlations observed between the single-interval y/n and the two-interval tasks (whole-head condition *m* = 0.89, *p* < 0.002; eye-region condition *m* = 1.01, *p* < 0.002), as can be seen from Figure 5B. Correlations between the single-interval l/r task and the other tasks were not significant in the whole-head condition (*p* = 0.45 and *p* = 0.65 for the single-interval y/n and two-interval tasks, respectively), but were evident in the eye-region condition (single-interval y/n *m* = 0.72, *p* = 0.002; two-interval *m* = 0.67, *p* = 0.002), as seen in Figure 5C.

The measurements from the single-interval l/r and the two-interval tasks were similar to the measurements from those tasks in Experiment 1, with a stronger repulsive effect in the single-interval l/r task compared to the two-interval task, and a stronger repulsive effect in both tasks in the eye-region condition compared to the whole-head condition. The large difference in measurements between the single- and two-interval tasks virtually disappeared when observers were asked to make a non-directional judgment in the single-interval task that was similar to the judgment made in the two-interval task. This strongly suggests that the difference between measurements was the result of the type of judgment required by each task: Observers are showing different effects of head orientation on perceived gaze direction depending on whether they are making a directional (left vs. right) or non-directional (concerning whether gaze is directed at them or not) judgment. There are several possible explanations for this; observers may be weighting the same sensory evidence differently across judgments, or they may be integrating different sensory cues to gaze direction according to the task at hand. The weaker correlation between measures across judgments (compared to within the non-directional judgment tasks) could suggest that observers are integrating different evidence across the judgments (as this would increase uncorrelated noise in the judgments). Thus, this possibility was examined in Experiment 3.

**FIGURE 6:**
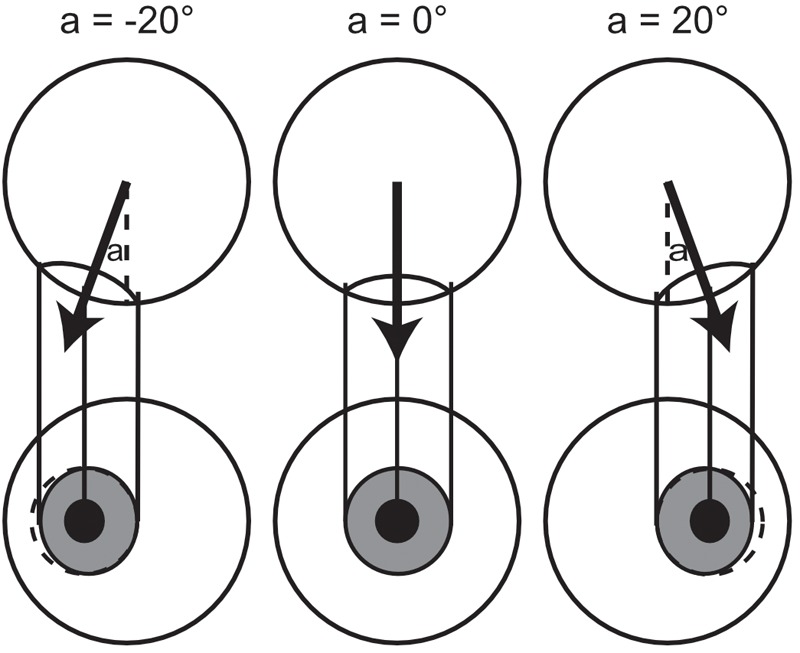
Change in the apparent circularity of the pupil/iris with eye rotation. The top row of circles shows a top-down look of an eyeball, to show the angular rotations. The bottom row shows the front view from the observer’s perspective, where the apparent horizontal extent of the iris/pupil is related to the relative rotation of the eye, and circularity is shown in the dotted outline. The aspect ratio (width to height), δ, of the ellipse projected to the observer can be related to the angular rotation of the eye relative to the observer, a, by trigonometry; δ = |cos(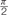 -a)|, assuming direct gaze has a deviation of 0°, equal but opposite deviations will produce the same apparent aspect ratio. An aspect ratio of 1 indicates direct gaze, and anything less than 1 indicates deviation from direct, horizontally in either direction.

The circularity of the pupil/iris offers a non-directional cue to direct gaze. When gaze is directed at the observer (irrespective of the orientation of the head) the shape of the pupil projected to the observer will be circular. As gaze deviates away from the observer, the projected shape of the pupil becomes more elliptical, but equally so in both leftward and rightward gaze directions, as shown in Figure 6. Thus, the apparent circularity of the pupil/iris could be used to assess whether gaze is direct or not, but offers no evidence as to whether gaze is deviated in a more rightward or leftward direction. Humans (and monkeys) are especially sensitive to the aspect ratio of ellipses, being capable of discriminating perfect circles from ellipses with an aspect ratio of just 0.98 (Laursen and Rasmussen, 1975). Thresholds for discriminating aspect ratios of ellipses are smaller than those for discriminating rectangles (Zanker and Quenzer, 1999; Morgan, 2005) and evidence suggests that this hyperacuity for regularity in circles is supported by specialized mechanisms for curvature discrimination (Dobbins et al., 1987; Dumoulin and Hess, 2007). This specialized sensitivity means that the circularity cue could be used to judge whether gaze is direct or averted, despite the fact it offers no evidence for deciding whether gaze is directed left or right.

Experiment 3 tests whether the circularity cue is used in non-directional judgments by comparing directional and non-directional judgments under two eye conditions: rotated and translated eyes. In natural conditions, when someone averts their eyes from an observer in the horizontal dimension, this causes two sensory transformations to the iris from the observer’s perspective. First, as described above, the pupil/iris is rotated, such that the shape it projects to the observer becomes more elliptical. Second, the position of the pupil/iris is translated within the visible eye region, such that it moves closer to the edge of the eye socket. In the rotated eye condition in Experiment 3 the direction of gaze is defined by the rotation of the eyes as in normal stimuli, where the position of the iris is translated and the projected shape becomes more elliptical, such that the circularity of the pupil/iris can be used as a cue for direct gaze (as in the previous experiments). However, in the translated eye condition, eye direction is defined by moving the pupil/iris such that it is centered in the same location as with rotated eyes, but the pupil/iris remains circular. Thus, in the translated eye condition the circularity of the pupil cannot be used to infer averted gaze, but there are still pupil translation cues to gaze direction. It is predicted that if the use of the circularity cue is driving the difference between directional and non-directional judgments, then there will be no difference between these judgments in the translated eye condition. Furthermore, if the circularity of the pupil/iris is used as a cue to direct gaze, then it is predicted that observers will be more willing to accept gaze as direct in the translated eye condition compared to the rotated eye condition, when making non-directional judgments, even in directly oriented heads.

## Experiment 3

### Methods

Methods were largely the same as in the previous experiments, with the following exceptions.

#### Participants

Twenty observers were recruited in the same manner as in Experiments 1 and 2. All participants gave written informed consent to participating. There was only one group of participants as only the whole-head stimuli were tested. After applying the same exclusion criteria as previously, 18 observers were included in the analysis.

#### Stimuli

In the rotated eye condition, the stimuli were exactly the same as presented in Experiments 1 and 2. In the translated eye condition, the manipulation of eye deviation was conducted by drawing a circular pupil and iris centered on the same point as in the rotated eye condition (as if filling in the dotted outline in the rotated eyes of Figure 6). An additional head orientation condition was added to the procedure, where stimuli with direct facing heads were created in the same manner as already described.

#### Procedure

Participants were asked to complete two tasks: a single-interval y/n task and a single-interval l/r task. On each trial in the single-interval l/r task, observers were presented with a stimulus and asked to judge whether gaze was directed to the left or to the right of them. Responses were entered by pressing ‘1’ for leftward and ‘2’ for rightward on a high-speed mechanical keyboard, which allowed for the accurate measurement of reaction times. Observers were presented with gaze deviations ranging from -14° and 14° degrees in steps of 2°, and observers made eight responses to each gaze deviation for each of the three head orientations (-15°, 0°, and 15°) and in each eye condition (translated or rotated), which were presented in pseudo-random order, making a total of 720 trials. These exact same trials were used in the single-interval y/n task, though presented in a different random order. The only difference was the judgment required of the observers: they were asked to press ‘1’ if they thought gaze was not directed at them, or ‘2’ if they thought gaze was directed at them. The order of tasks was randomized across participants, and both tasks were completed in a single session of approximately 1 h.

#### Analysis

Analysis was conducted in the same manner as Experiments 1 and 2 for the rotated and translated eye conditions separately. After calculating the PSEs, two observers were removed from further analysis as they displayed PSEs beyond the range of eye deviations presented in the l/r task, and closer inspection indicated that their responses did not vary with gaze direction, even at the most extreme eye deviations.

The influence of head orientation on perceived gaze direction was then compared across tasks and conditions, along with measurements of subjectively direct gaze in the direct head. Two follow-up exploratory analyses were then conducted. The first examined the effect of eye rotation vs. translation on the tendency of observers to respond that gaze was direct. The other explored measures of participants’ reaction times. Reaction times were measured relative to the offset of the stimulus, and for each comparison the median reaction time of each observer was used for further analysis.

### Results

**FIGURE 7:**
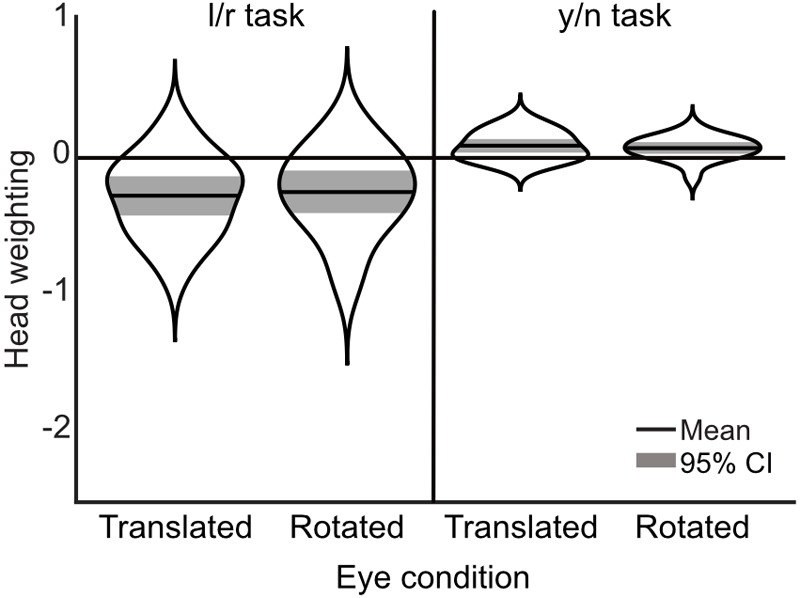
Measurements of head weighting by task and condition from Experiment 3. The means are shown in the solid black lines, the width of each ‘violin’ corresponds to the probability density, and the colored shaded area marks the 95% confidence interval. A negative weighting indicates an overall repulsive effect of the head.

A 2×2 repeated measures ANOVA with task (l/r vs. y/n) and eye condition (rotated vs. translated) as within subjects factors, revealed a significant effect of task [*F*(1,17) = 51.395, *p* < 0.001, $\eta_{p}^{2}$ = 0.751] on the influence of head orientation on perceived gaze direction. There was no effect of eye condition [*F*(1,17) = 0.004, *p* = 0.949, $\eta_{p}^{2}$ < 0.001], and no interaction [*F*(1,17) = 1.723, *p* = 0.207, $\eta_{p}^{2}$ = 0.092]. For comparison with previous experiments, the corresponding head weightings are shown in Figure 7. The average measured head weightings are, if anything, less repulsive than previously measured in the left/right task [*M*(rotated) = -0.27 and *M*(translated) = -0.25, compared to *M* = -0.35 in Experiment 2], and in the yes/no [*M*(rotated) = 0.09 and *M*(translated) = 0.07, compared to *M* = -0.002 in Experiment 2], though no formal comparisons have been made.

In the y/n task, observers accepted a greater range of gaze deviations as being directed toward them in the rotated eye condition compared to the translated eye condition, as measured by the separation between the two logistic functions describing the proportion of direct responses, which is a similar measure to the width of the cone of direct gaze (Mareschal et al., 2013) [mean difference = 0.764, *t*(17) = 2.983, *p* = 0.008]. We had hypothesized the effect to be in the opposite direction. A comparable measure in the l/r task is the distance from 25% rightward responses to 75% rightward responses, based on the fitted logistic function. There was no significant difference in this corresponding width measure between translated and rotated eye conditions in observers’ l/r responses to the direct head [mean difference = 0.404, *t*(17) = 0.717, *p* = 0.483].

**FIGURE 8:**
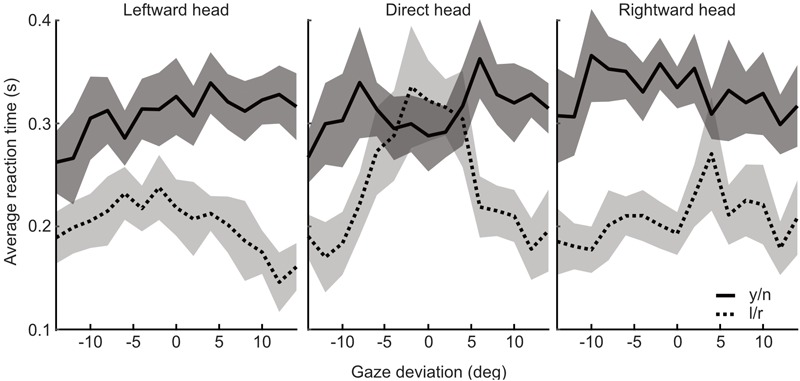
Analysis of reaction times from Experiment 3. Reaction times (seconds) plotted against gaze deviation in the leftward, direct, and rightward oriented heads for the yes/no (solid/dark) and left/right (dotted/light) tasks, respectively. Shaded regions indicate 95% within subject confidence intervals.

Median reaction times were found to be significantly shorter in the l/r task compared to the y/n task [mean difference = -0.1s, *t*(17) = -2.898, *p* = 0.01]. The pattern of reaction times across gaze deviations within each task was then examined. In the l/r task reaction times tended to increase with decreasing gaze deviation, whereas, in the y/n task, reaction times were seen to increase with increasing gaze deviation up to about ±6 degrees, and then decrease again with increasing deviations. After separating the median reaction times by head orientation it was found that the distinct patterns of response times was driven by the direct head condition (Figure 8). Whilst this pattern may have been washed out by variability in observers’ perceived direct gaze in the rotated heads, it is also unlikely that this pattern is driving the overall differences in reaction times between tasks, as the differences within the y/n and l/r tasks are smaller than the differences between them.

If the use of the circularity cue were causing the difference between the directional and non-directional judgments, then this difference would be (at least) diminished in the translated eye condition. We found no evidence for this. Rather, differences in measurements of the influence of head orientation on perceived gaze direction were driven solely by task differences. This suggests that the use of the circularity cue is not driving the difference between the directional and non-directional tasks, and there is little interaction between this cue and the effect of the orientation of the head on perceived gaze direction.

It could be that, by intermixing the translated and rotated eye conditions, observers stopped relying on the circularity cue altogether as it became unreliable. If this were the case, and the use of the circularity cue were driving the task differences, then there would be no difference in the measured influence of head orientation on perceived gaze direction across tasks, but the task differences were just as apparent as in Experiment 2. Furthermore, if observers were not using the circularity cue at all, then there should be no difference in measurements across eye conditions. Yet, there was a significant increase in the width of the cone of direct gaze measured in the y/n task in the rotated eye condition compared to the translated eye condition. It was expected that, if observers were using the circularity of the pupil as a cue to direct gaze, then observers may show a wider cone of direct gaze in the translated eye condition, where the pupil remains circular across gaze deviations. That the opposite effect was observed suggests that the difference was the result of the change in the geometric and luminance cues within the eye region: In the translated eye condition, the pupil/iris was wider than in the rotated eye condition when the eyes were deviated, meaning that the edge of the iris was closer to the edge of the eye when the centers of the pupils were fixed (as shown in Figure 6). The distance of the edge of the iris to the edge of the eye, or perhaps more simply, the slight change in the apparent sclera ratio this would create, could be taken as evidence for eye deviation, causing the decrease in the width of the cone of direct gaze.

The difference in reaction times between tasks could be the result of a difference in the accumulation and processing of sensory evidence between tasks. One proposal is that there may be a difference in the processing of information from an allocentric (with respect to 3D space) compared to an egocentric (with respect to oneself) perspective. For example, Senju and Johnson (2009) have proposed a model in which direct gaze is initially processed by a subcortical route via the superior colliculus, pulvinar, and amygdala. This fast route utilizes mainly low spatial frequencies and is proposed to modulate the cortical processing of more finely tuned gaze direction information that feeds through to the anterior Superior Temporal Sulcus (aSTS). The difference in the processing times of these pathways could be responsible for the difference in reaction times associated with each judgment, though another possibility is that the difference in reaction times emerged at the response mapping stage. These two possibilities were tested in Experiment 4, where the left/right and yes/no tasks were compared over two stimulus presentation duration conditions. By presenting stimuli for very short or relatively long durations the amount of evidence accumulation is restricted and increased, respectively. If the difference between the tasks results from a difference in the amount of evidence accumulation required for each decision, then we will observe an interaction between presentation duration and the difference in the head weighting between the two tasks.

## Experiment 4

### Methods

Methods were the same as used in the previous experiments with the exceptions detailed below.

#### Participants

Twenty-five participants were recruited in the same manner as before. All participants gave written informed consent to participating. As in Experiment 3, only the whole head stimuli were tested, in a single group of participants. After applying the same exclusion criteria as in previous experiments, 17 participants were included in the final analysis.

#### Procedure

Participants were asked to complete the left/right and yes/no tasks, using the same stimuli and task instructions as in Experiment 2. There were two changes to the experimental design. First, the response keys were changed such that, in the left/right task observers were asked to press the ‘j’ key for ‘left’ and the ‘k’ key for ‘right,’ and in the yes/no task, observers pressed ‘j’ for ‘direct’ and ‘k’ for ‘not direct’ (note that the order of these yes/no responses on the keyboard is reversed compared to Experiment 3). Second, two stimulus duration conditions were included: in previous experiments stimuli were presented for 400 ms (including ramp), here, in the ‘short duration’ condition, stimuli were presented for a total of 150 ms, and in the ‘long duration’ condition, stimuli were presented for a total of 900 ms, and each of these conditions included a 50 ms cosine ramp at stimulus onset and offset. Observers were encouraged to respond as quickly but as accurately as they could after the stimulus. Observers completed the four experimental conditions (2 tasks × 2 duration conditions) over four separate blocks, in pseudo-randomized order.

### Results

**FIGURE 9:**
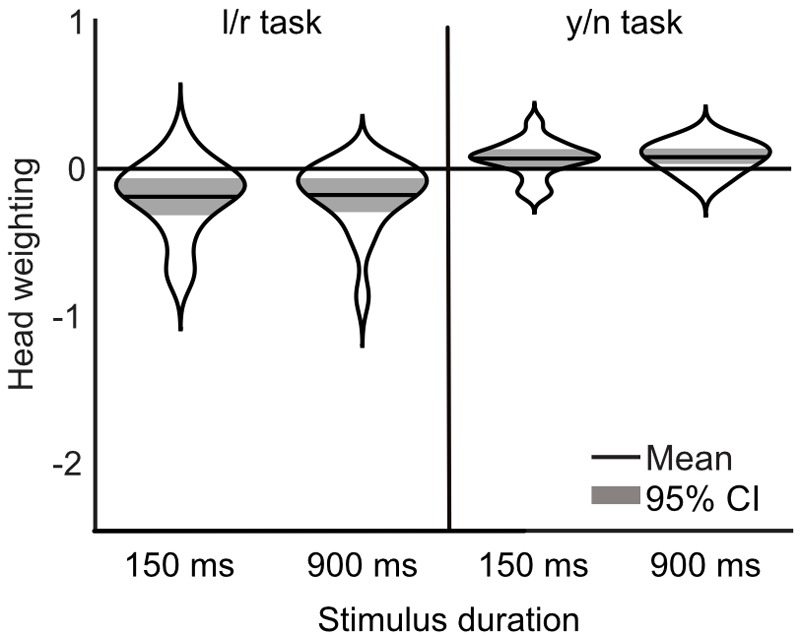
Measurements of head weighting by task and condition from Experiment 4. The means are shown in the solid black lines, the width of each ‘violin’ corresponds to the probability density, and the colored shaded area marks the 95% confidence interval. A negative weighting indicates a net repulsive effect of the head.

**FIGURE 10:**
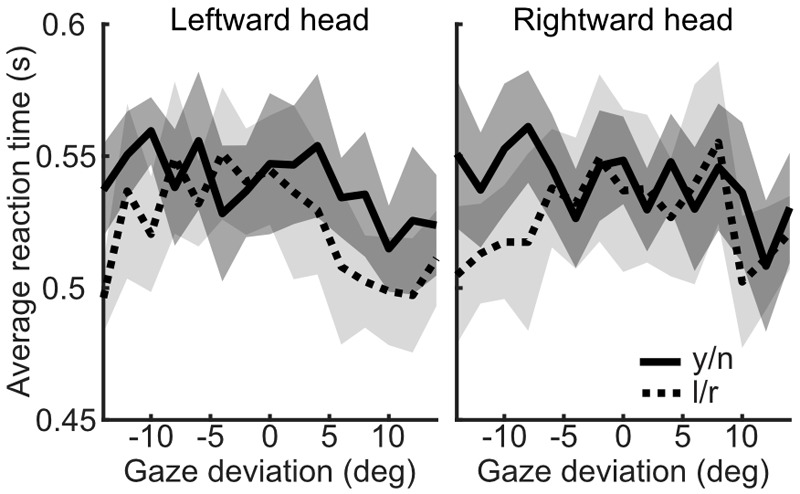
Reaction times by task, across head orientations and gaze deviations, from Experiment 4. The yes/no task is shown in the solid line (mean) with the dark shaded region (95% within subject confidence interval), whilst the left/right task is shown in the dotted line (mean) with light shaded region (95% CI).

The influence of head orientation on perceived gaze direction was calculated for each task and each stimulus duration condition separately. These measures, transformed into weightings of head orientation, are presented in Figure 9. A 2×2 repeated measures ANOVA, with task (l/r vs. y/n) and stimulus duration (150 ms vs. 900 ms) as within subjects factors revealed a significant effect of task [*F*(1,16) = 34.81, *p* < 0.001] but no significant effect of stimulus duration [*F*(1,16) = 0.02, *p* = 0.89] and no significant interaction [*F*(1,16) = 0.02, *p* = 0.88]. Figure 10 shows the same comparison of reaction times as in Experiment 3, for each stimulus duration: there was clearly no effect of task on reaction time.

These results show that stimulus duration had no significant effect on the measures of the influence of head orientation on perceived gaze direction, suggesting that any differences in the time course of accumulation and processing of sensory evidence do not significantly contribute to the differences between judgments. Furthermore, the difference in reaction times between judgments found in Experiment 3 was not replicated in Experiment 4. Thus, the difference in reaction times was likely a difference at the response mapping stage. In Experiment 3 observers were asked to press ‘1’ if gaze was averted and ‘2’ if gaze was direct, whereas, in Experiment 4, observers were asked to press ‘j’ if gaze was direct and ‘k’ if gaze was averted (the reverse order on the keyboard). It may be that this order of judgments feels more natural to the observer and enables them to make yes/no responses as fast as they make the left/right responses. Together, these results indicate that there is little evidence for a difference in sensory evidence accumulation between tasks. It is therefore unlikely that the difference between tasks is the result of observers utilizing different sources of sensory evidence to make their judgments in each task.

## Discussion

In testing a bias minimizing measure of the influence of head orientation on perceived gaze direction, we (Balsdon and Clifford, 2017) found a large difference between the measures of a bias minimizing two-interval task and a single-interval task that was similar to previous tasks. Experiment 1 tested whether the difference between the single- and two-interval tasks could arise from a superficial difference in the way stimuli were presented. Any effect of apparent motion cues (which could have been more prominent in the two-interval compared to the single-interval task) was minimized by jittering the location of the stimuli on the screen, and adding noise after each stimulus presentation. We also tested the possibility that the perceived head orientation of the second stimulus was influenced by the first, by testing a two-interval task where the same head orientation was presented twice. There was no difference in measures from the two-interval task with same- and oppositely oriented heads. Furthermore, recent evidence did not find a sequential effect of head orientation (where the reported head orientation may be influenced by previously presented head orientations; Alais et al., 2018). Together, this evidence suggests that the difference between tasks cannot be reduced to a superficial difference in the way stimuli are presented.

Experiment 2 provided evidence that the difference between tasks resulted from something more enduring than differences in stimulus presentation. When observers judged whether gaze was direct or averted in a single-interval y/n task, the measured influence of head orientation on perceived gaze direction matched that of the two-interval task (both in terms of the group average, and the correlation between individual measures), despite the fact that observers in the single-interval y/n task were presented with exactly the same stimuli, in the same manner, as in the single-interval l/r task. In comparison, measurements between the two types of judgments (directional vs. non-directional) differed systematically, where the single-interval l/r task revealed a stronger net repulsive effect in both the whole-head and eye-region conditions compared to when observers made a non-directional judgment. Although observers who displayed a stronger repulsive effect in the directional judgment task also tended to display a stronger repulsive effect in the non-directional judgment tasks, the correlations between measures across these types of judgments were far weaker than the correlation between the two tasks with non-directional judgments (the single-interval y/n task and the two-interval task). This could suggest that observers were using different evidence across the two judgments.

The circularity of the pupil/iris could be used to make non-directional judgments of gaze direction, but not directional judgments. Removing the circularity cue (by keeping the pupil/iris circular and translating the pupil) in Experiment 3 did not affect measurements of the influence of head orientation on perceived gaze direction. There was some effect of the circularity cue on perceived gaze direction, as observers were more likely to respond that gaze was averted in the translated eye condition, suggesting that the experimental manipulation did have some effect on perceived gaze direction, just not on the influence of head orientation on perceived gaze direction. Although this is the only cue (to our knowledge) that could be integrated differently between directional and non-directional judgments, the difference in reaction times between tasks in Experiment 3 suggested that there could still be some difference in the way that observers accumulate and process sensory information for gaze direction across judgments (for example, in utilizing a subcortical route in processing information relevant to whether gaze is direct).

Experiment 4 tested whether the difference in reaction times reflected a difference in stimulus processing between the two judgments, or a difference at the response mapping stage. If a there was a difference in the accumulation and processing of sensory evidence depending on the judgment, and this was causing the difference in the measures of the influence of head orientation on perceived gaze direction, then we would have expected the difference in the measurements between judgments to change with the stimulus duration. There was no effect of stimulus presentation duration on the measures of the influence of head orientation on perceived gaze direction and, importantly, no interaction between stimulus duration and judgment type/task. Rather, when the correspondence between response buttons and response was reversed in the y/n task in Experiment 4, the large difference in reaction times seen in Experiment 3 was no longer evident, suggesting that the reaction time differences seen in Experiment 3 were the result of a difference at the response mapping stage. Thus the results of Experiment 4 do not show any evidence, at the behavioral level, that the difference between measures of the influence of head orientation on perceived gaze direction from directional and non-direction judgments is caused by differences in evidence accumulation and processing, such as the utilization of a fast subcortical route for the prioritized detection of direct gaze. This, together with the evidence of Experiments 1–3, suggests that observers are weighting the same perceptual evidence differently depending on their intention in examining gaze stimuli – whether they are going to make a directional or non-directional judgment.

When asked to make a directional judgment about where someone is looking, the orientation of the head has an overall repulsive effect on the perceived direction of gaze (Anstis et al., 1969; Otsuka et al., 2014, 2015, 2016). This net repulsive effect results from a stronger weighting of the indirect effect of head turn on the eye-region, compared to the direct attractive effect of head orientation (in accordance with the dual-route model, Figure 1). When the direct attractive effect is weakened (for example, when showing only the eye-region of stimuli) this repulsive effect increases, and gaze direction is perceived even more in the opposite direction to the orientation of the head.

When observers were asked to make non-directional gaze judgments, their responses suggested that their perception was closer to the veridically presented gaze deviation than in the directional judgment task. It is not the case that there is no influence of head orientation on perceived gaze direction in the non-directional judgment task, as responses in the eye-region condition still indicated a net repulsive effect. Rather, the incorporation of the head as a direct cue in the whole-head condition appears to balance the repulsive effect of head rotation on the eye-region information, allowing for a high level of gaze constancy (invariance in perceived gaze direction across different head orientations; Otsuka et al., 2015). This balance is lost when the direct cue is weakened in the eye-region condition, as the information about head orientation is much impoverished.

It should be noted that not all previous experiments using a non-directional judgment have found near perfect gaze constancy. Gibson and Pick (1963; replicated by Moors et al., 2016) found a net repulsive effect of head orientation when observers reported whether the gaze of a real human ‘looker’ was directed at them or not. We calculated the head weighting in Gibson and Pick’s data to be around -0.1 (on average, 2.8 degrees gaze deviation in the same direction as head orientation was perceived to be direct in heads rotated 30 degrees), which is a far smaller net repulsive effect compared to that measured by the directional judgment tasks presented here and previously (an average head weighting of -0.25). The main differences between the non-directional judgment tasks presented here and that of Gibson and Pick (1963) are the angle of head rotation (30 degrees compared to the 15 degrees here), the viewing distance (observers were 2 m from the face in Gibson and Pick), and the viewing time (observers viewed the face until they gave a response). This may mean that at greater distances, or with increased head rotation, observers weight the sensory evidence differently when making non-directional judgments, and thus show a different balance of the attractive and repulsive effects of head orientation. These possibilities deserve further investigation. Another possibility, discussed in Moors et al. (2016) is that there can be some inter-individual variability in the effect of head orientation on perceived gaze direction. Gibson and Pick (1963) included just six participants; Moors et al. (2016) included twelve, whereas our experiments show consistent replication with sample sizes of 20. Indeed, the violin plots across all experiments show observers display a distribution of effects that crosses 0 in every task.

The two-interval task was originally tested as a method of minimizing bias in the measurement of the effect of head orientation on perceived gaze direction (Balsdon and Clifford, 2017), as this would be useful in comparing gaze perception across populations that may systematically differ in their response biases, such as in clinical populations. The two-interval task produces measurements significantly different from the single-interval l/r task, and the measured effect in the two-interval task does not seem to reflect the immediate impression that dominates our perception when simply viewing example stimuli, such as those in Figure 2. Given that the two-interval task does not measure the same effect of head orientation on perceived gaze direction as ‘directional’ tasks such as when observers are making left/right categorizations of gaze, a more appropriate task for testing clinical populations may therefore be the use of an estimation judgment, as in Anstis et al. (1969) and Otsuka et al. (2016). Such judgments are likely to be less affected by response bias, but still measure the overall repulsive effect that dominates passive viewing. This is not to say that measurements from the two-interval task, or non-directional judgment tasks *per se*, do not reflect the perceptual experience of the observer. What the results of these experiments suggest is that when tasked with deciding if they are being looked at, observers weight and integrate sensory evidence differently, in such a way that there is a genuine (though slight) difference in their perceptual experience of gaze direction. Under the dual-route model this can be captured simply by a change in the relative weightings of the direct and indirect effects of head orientation on perceived gaze direction.

Flexibility in the weighting of sensory cues integrated into the perception of high-level visual stimuli, such as gaze direction, has been demonstrated across a number of previous experiments in which the stimulus was directly manipulated. For example, Langton et al. (2004) showed similar effects of head orientation on perceived gaze direction irrespective of whether the orientation of the head was cued by the angle of the nose or the contour of the head, suggesting that observers are capable of extracting the orientation of the head from whichever sensory cue was provided. Noll (1976), and more recently Otsuka and Clifford (2017), found that observers could use sensory information from either eye when the other was not visible but would rely more heavily on information from the further eye when both were visible. This suggests that observers are more than capable of utilizing whatever sensory evidence is available for the perception of another’s gaze direction, and integrating this evidence in a flexible manner. However, the current study provides the first evidence that this flexible integration is important not only for dealing with large changes in the quality and availability of sensory evidence, but also for the intention of the observer when accumulating sensory evidence – in this case, whether they are to make a directional or non-directional judgment. This clearly has strong implications for future research into the perception of gaze direction, and high-level vision in general.

One characterization of the difference in the intention of the observer across the directional and non-directional tasks is that observers take an allocentric vs. egocentric perspective, also termed triadic vs. dyadic gaze perception. Egocentric/dyadic gaze perception involves examining where the looker is directing their gaze relative to the observer, whilst allocentric/triadic gaze perception involves examining where the looker is directing their gaze with respect to the world. Whilst egocentric gaze perception is important for interpersonal relations (Senju and Johnson, 2009; Hamilton, 2016) and has been linked with both neural reward circuitry and threat circuitry (Kampe et al., 2001; Adams et al., 2003), allocentric gaze perception is important for joint attention to objects in the environment and the capture of attention through gaze cueing (Friesen and Kingstone, 1998). These two roles of gaze perception could be thought to require different emphasis on different cues, and indeed, it was the adoption of an egocentric perspective that Seymour et al. (2017) found to cause a difference in gaze processing in patients with schizophrenia. It would be interesting, given the emphasis on local vs. global processing in people with Autism Spectrum Disorder (Simmons and Todorova, 2018), to examine to what extent the weighting of head orientation information changes across allocentric and egocentric perspectives in this population, for example, by comparing responses across directional and non-directional judgment tasks.

## Conclusion

In these experiments, we showed systematic differences in the measured effect of head orientation on perceived gaze direction, depending on the task of the observer. We showed that these differences were not caused by some artifact of stimulus presentation (Experiment 1), but rather, a difference in the type of judgment being made – whether observers were judging the horizontal direction of gaze, or making a non-directional judgment about whether they were being looked at (Experiment 2). The difference between judgments is not because observers are relying on different sensory evidence depending on the judgment (Experiments 3 and 4), but because observers are integrating the same evidence in a flexible manner; applying a different relative weighting to the direct head cue and information from the eye region according to their intention in sampling evidence of gaze direction. This is important for future research in considering what type of task to implement for testing different hypotheses, especially in research with clinical populations where systematic differences in the ways patients conceptualize tasks may contribute to differences in measures.

## Data Availability

The raw data supporting the conclusions of this manuscript will be made available by the authors, without undue reservation, to any qualified researcher.

## Author Contributions

TB and CC designed the experiments and analyses. TB carried out the experiments, conducted the analyses, and wrote the manuscript. CC contributed to the manuscript and provided equipment. Both authors gave final approval for publication.

## Conflict of Interest Statement

The authors declare that the research was conducted in the absence of any commercial or financial relationships that could be construed as a potential conflict of interest.

## Footnotes

1 http://www.daz3d.com/
